# A Genetically Encoded Bioluminescence Intracellular Nanosensor for Androgen Receptor Activation Monitoring in 3D Cell Models

**DOI:** 10.3390/s21030893

**Published:** 2021-01-29

**Authors:** Maria Maddalena Calabretta, Antonia Lopreside, Laura Montali, Luca Cevenini, Aldo Roda, Elisa Michelini

**Affiliations:** 1 Department of Chemistry “Giacomo Ciamician”, University of Bologna, 40126 Bologna, Italy; maria.calabretta2@unibo.it (M.M.C.); antonia.lopreside2@unibo.it (A.L.); laura.montali2@unibo.it (L.M.); luca.cevenini5@unibo.it (L.C.); aldo.roda@unibo.it (A.R.); 2 Center for Applied Biomedical Research (CRBA), University of Bologna, 40126 Bologna, Italy; 3 Istituto Nazionale di Biostrutture e Biosistemi (INBB), 00136 Rome, Italy; 4 Health Sciences and Technologies-Interdepartmental Center for Industrial Research (HST-ICIR), University of Bologna, Ozzano dell’Emilia, 40064 Bologna, Italy

**Keywords:** bioluminescence, luciferase, complementation assay, 3D cell model, androgen receptor

## Abstract

In recent years, there has been an increasing demand for predictive and sensitive in vitro tools for drug discovery. Split complementation assays have the potential to enlarge the arsenal of in vitro tools for compound screening, with most of them relying on well-established reporter gene assays. In particular, ligand-induced complementation of split luciferases is emerging as a suitable approach for monitoring protein–protein interactions. We hereby report an intracellular nanosensor for the screening of compounds with androgenic activity based on a split NanoLuc reporter. We also confirm the suitability of using 3D spheroids of Human Embryonic Kidney (HEK-293) cells for upgrading the 2D cell-based assay. A limit of detection of 4 pM and a half maximal effective concentration (EC_50_) of 1.7 ± 0.3 nM were obtained for testosterone with HEK293 spheroids. This genetically encoded nanosensor also represents a new tool for real time imaging of the activation state of the androgen receptor, thus being suitable for analysing molecules with androgenic activity, including new drugs or endocrine disrupting molecules.

## 1. Introduction

The availability of reliable tools for the rapid detection of protein–protein interactions (PPI) has become necessary for identifying biologically active compounds, especially in the early phases of drug discovery [1,2]. Among the different methods that are available, split proteins have provided an elegant way to detect and image PPI in real time [3]. A reporter protein can be split into two fragments that can spontaneously reassemble, without covalent linking, to reconstitute the functional protein. Therefore, the cDNAs encoding for the two fragments can be genetically fused to produce two genes encoding for the target proteins under investigation; these genetically encoded sensors can be inserted into living cells and, after interaction of the two target proteins, functional reassembly of the split protein will occur [4,5].

A pivotal work, describing the exploitation of split reporters in protein-fragment complementation assays (PCA) for targeting PPI, was reported by Johnsson and Varshavsky with a split ubiquitin [6]. Since then, several reporter proteins have been used in PCA, such as the Green Fluorescent Protein (GFP) and its variants [7], B-galactosidase [8], and different luciferases [9,10,11]. One of the main limitations of split reporters is that they rely on nonspecific reassembly of the fragments in the absence of an interaction between the target proteins and on irreversible protein complementation. To circumvent these issues, Dixon et al. developed a new complementation reporter, called NanoLuc Binary Technology (NanoBiT), starting from the engineered luciferase NanoLuc derived from a deep-sea luminous shrimp. NanoLuc luciferase is characterized by an enhanced bioluminescence, making it an excellent reporter for cell-based assays [12]. NanoBiT has two subunits: A large one (LgBiT) and a small peptide one (SmBiT), of 18 and 1.3 kDa, respectively, characterized as having a very low affinity. Notably, the reassembly is reversible, thus enabling both protein association and dissociation to be monitored [3]. NanoBiT has been successfully applied to monitor PPI and detect intracellular calcium levels [13], endosome disruption [14], and circulating microRNAs with such a high sensitivity that it could be implemented in smartphone-based assays [15]. In another configuration, split reporter proteins provide a single-molecule probe to monitor ligand-induced conformational changes in a single molecular backbone [16,17,18]. An elegant modular sensor was developed by Ni et al., based on competitive intramolecular complementation of split NanoLuc luciferase, allowing tuning of the sensor’s dynamic range [19]. Folding sensors relying on human estrogen receptor ligand-binding domain (hER-LBD) fusion proteins leading to split Renilla/firefly luciferase reporter complementation were particularly successful in detecting estrogens and antiestrogens both in cell cultures and for the in vivo imaging of ligand-induced intramolecular folding in living mice [18]. Analogously, the human androgen receptor binding domain (hAR-LBD) was sandwiched between a dissected click beetle luciferase [16]. The androgen receptor is a ligand-activated nuclear transcription factor that is responsible for mediating the intracellular action of androgens [20]. It is involved in the maintenance and function of male reproductive organs and plays a key role in prostate cancer and other pathologies. Since its role is exerted via homodimerization and interactions with cofactors, PCA was proposed for screening adverse chemicals acting on the androgen receptor and new therapeutic targets [16].

All these assays are generally performed either in vitro using purified chimeric proteins or with conventional two-dimensional (2D) cell cultures. Although these cell models are still considered the “gold standard”, a growing body of evidence suggests that they fail to replicate the in vivo complexity and actual cellular architecture comprising the extracellular matrix (ECM) microenvironment and cell–cell interactions [21].

3D cell culture systems overcome many of the limitations of traditional 2D cell culture systems, more closely mimicking the complex phenotypic heterogeneity that chemical gradients produce during cell growth. In fact, 3D cellular systems reproduce cell–cell and cell–matrix interactions, intra- and inter-cellular signaling networks, and diffusion/transport conditions, which are important for differentiation, proliferation, and various cellular functions. We previously demonstrated the suitability of using bioluminescence (BL) as a detection technique for reporter assays developed in 3D cell models from human cell lines [22].

**Figure 1:**
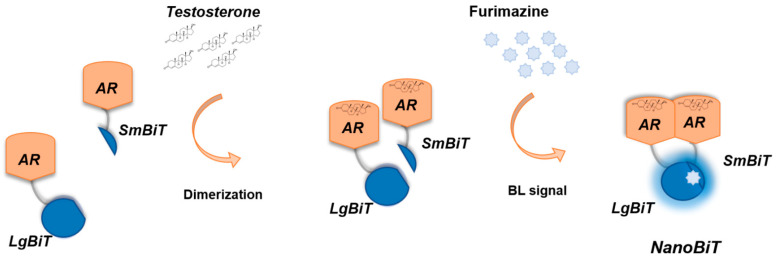
Schematic representation of the genetically encoded nanosensor principle. A large NanoLuc Binary Technology (NanoBiT) subunit (LgBiT) and small NanoBiT subunit (SmBiT) are genetically fused to the human androgen receptor (hAR), in the presence of testosterone, used as a model ligand with androgenic activity. Dimerization of hAR causes the reconstitution of NanoBiT luciferase, which in turn will emit light after the addition of the furimazine substrate.

We hereby report an intracellular nanosensor for androgenic activity detection relying on the human androgen receptor (hAR) fused to NanoBiT that can be employed in 2D and 3D cell models (Figure 1). Human Embryonic Kidney (HEK-293) cells were transiently transfected with vectors expressing the two chimeric proteins LgBiT-hAR and hAR-SmBiT under the control of a constitutive promoter. This genetically encoded sensor also represents a new tool for real time imaging of the activation state of the androgen receptor, thus being suitable for analysing molecules with androgenic activity, including new drugs or endocrine disrupting chemicals.

## 2. Materials and Methods

### 2.1. Reagents and Plasmids

Human embryonic kidney HEK293 cells were obtained from the American Type Culture Collection (ATCC, Manassas, VA, USA), and penicillin, streptomycin, and all cell culture reagents were obtained from Carlo Erba Reagents (Cornaredo, Milano, Italy). Enzymes for PCR and the cloning procedure (FastDigest, FastDigest Green Buffer, FastAP Thermosensitive Alkaline Phosphatase, T4 DNA ligase and Phusion high-fidelity DNA polymerase) were obtained from ThermoFisher Scientific (Waltham, MA, USA). The reporter vectors pGL3-SV40-LgBiT-hAR and pGL3-SV40-hAR-SmBiT were obtained by standard molecular cloning procedures. The PureYieldTM Plasmid Miniprep System kit, gel, and PCR extraction kit were obtained from Promega (Promega, Madison, WI, USA). The Nano-Glo^®^ Live Cell Assay System substrate and FuGENE^®^ HD transfection reagent were obtained from Promega (Promega, Madison, WI, USA). The 96-well microspace round-bottom cell culture plates were obtained from Elplasia (Kuraray, Tokyo, Japan). Testosterone and all other chemicals were purchased from Sigma-Aldrich (St. Louis, MO, USA).

### 2.2. Plasmids Construction

Standard molecular biology techniques were used to construct plasmids driving the constitutive expression of chimeric proteins hAR-SmBiT and LgBiT-hAR under different promoters. The hAR-SmBiT and LgBiT-hAR sequences were kindly provided by Promega (Promega, Madison, WI, USA). The herpes simplex virus (HSV) thymidine kinase (TK) promoter and early promoter of the simian virus 40 (SV40) were selected for constitutive expression in mammalian cells using pGL3 (SV40) and pTK vectors. hAR-SmBiT and LgBiT-hAR sequences were amplified by a polymerase chain reaction (PCR) and cloned using NheI and SalI fast digestion enzymes. The developed mammalian expression vectors were called pTK-hAR-SmBiT, pTK-LgBiT-hAR, pGL3 (SV40)-hAR-SmBiT, and pGL3 (SV40)-LgBiT-hAR. All vector sequences were confirmed by restriction analysis and sequencing (Supplementary Materials Figure S1).

### 2.3. Comparison of TK and SV40 Promoters

To evaluate the expression of chimeric proteins hAR-SmBiT and LgBiT-hAR under the regulation of different promoters (TK and SV40) and to define the optimal co-transfection conditions and incubation times, HEK293 cells, cultured using Dulbecco’s Modified Eagle’s Medium (DMEM) supplemented with 10% (*v*/*v*) FBS, 2 mM L-glutamine, 50 U/mL^−1^ penicillin, and 50 µg/mL^−1^ streptomycin, were plated in flat-bottom clear 24-well plates at a density of 8.0 × 10^4^ cells/well with 500 µL of complete growth medium and incubated under standard conditions for 24 h (37 °C, 5% CO_2_). After overnight incubation, medium was removed from the cells, washed with PBS 0.1 M pH 7.2, and replaced with DMEM fresh medium containing 10% (*v*/*v*) FBS charcoal stripped, 2 mM/L-glutamine, 50 U/mL^−1^ penicillin, and 50 µg/mL^−1^ streptomycin. Following this, HEK293 cells were transiently co-transfected with 0.25 µg pTK-hAR-SmBiT and 0.25 µg pTK-LgBiT-hAR, using the FUGENE^®^ HD standard protocol with a ratio of 3:1. In parallel, HEK293 cells were co-transfected with 0.25 µg of the pGL3-SV40-LgBiT-hAR construct and 0.25 µg of pGL3-SV40-hAr-SmBiT using the FUGENE^®^ HD standard method with a ratio of 3:1.

BL measurements were carried out after 24 and 48 h post-transfection. In particular, HEK293 cells were washed with PBS 0.1 M pH 7.2, detached with Trypsin 1X, and centrifugated for 5 min at 1200 rpm. After resuspension with PBS 0.1 M pH 7.2, a 87.5 µL volume of cell suspension (5.0 × 10^4^ cells) was seeded in a 96-well black microplate. The basal signal was measured for 1 min using Tecan Microplate Reader Spark^®^ (Tecan Trading AG, Männedorf, Switzerland) and the basal BL activity was measured for 3 min after the addition of 25 µL/well of 5× Nano-Glo^®^ Live Cell Assay System substrate. Finally, cells were treated with 12.5 µL of testosterone solution (final concentration of 100 nM) prepared in ethanol solution 1% (*v*/*v*) or with 12.5 µL of ethanol solution 1% (*v*/*v*) as a control. BL measurements were performed in real time with Tecan Microplate Reader Spark^®^, acquiring BL signals for 100 min.

### 2.4. 2D Cell Culture, Transfection, and PPI Assay

The HEK293 cell line was cultured using DMEM supplemented with 10% (*v*/*v*) FBS, 2 mM/L-glutamine, 50 U/mL^−1^ penicillin, and 50 µg/mL^−1^ streptomycin. The day before transfection, cells were plated in flat-bottom clear 24-well plates at a density of 8.0 × 10^4^ cells/well with 500 µL of complete growth medium and incubated under standard conditions for 24 h (37 °C, 5% CO_2_). After overnight incubation, medium was removed from the cells, washed with PBS 0.1 M pH 7.2, and replaced with DMEM fresh medium containing 10% (*v*/*v*) FBS charcoal stripped, 2 mM/L-glutamine, 50 U/mL^−1^ penicillin, and 50 µg/mL^−1^ streptomycin. Afterwards, HEK293 cells were transiently co-transfected with 0.25 µg of the pGL3-SV40-LgBiT-hAR construct and 0.25 µg of pGL3-SV40-hAR-SmBiT using the FUGENE^®^ HD standard method with a ratio of 3:1. At 48 h post-transfection, HEK293 cells were washed with PBS 0.1 M pH 7.2, detached with Trypsin 1X, centrifugated for 5 min at 1200 rpm, and resuspended with PBS 0.1 M pH 7.2. A volume of 87.5 µL of cell suspension containing 5.0 × 10^4^ cells was seeded per well in a 96-well black microplate. The basal signal was measured for 1 min by Tecan Microplate Reader Spark^®^ and the basal BL activity was measured for 3 min after the addition of 25 µL/well of 5× Nano-Glo^®^ Live Cell Assay System substrate. Finally, cells were treated in triplicate with 12.5 µL of testosterone solutions (concentration range from 0.001 to 100 nM) prepared in ethanol solution 1% (*v*/*v*) or with 12.5 µL of ethanol solution 1% (*v*/*v*) as a control. BL measurements were performed in real time with Tecan Microplate Reader Spark^®^, acquiring BL signals for 100 min. The correct BL signal was obtained by subtracting the basal signal of each well from its BL signal, while the testosterone dose-response curve was generated using GraphPad Prism software normalized to the BL signal of the control. All measurements were performed in triplicate and repeated at least three times. The detection limit is defined as the testosterone concentration that corresponds to the blank plus three times the standard deviation (s.d.).

The half maximal effective concentration (EC_50_), which is the concentration of testosterone which produces 50% of the maximum possible response, was calculated using the following equation: Y = Bottom + (Top-Bottom)/(1 + 10^((LogEC50-X) * Hillslope)), where X is the logarithmic concentration of testosterone, Y is the response, and Y starts at the Bottom and goes to the Top with a sigmoid shape. Data were analyzed using GraphPad Prism software.

### 2.5. 3D Cell Culture, Transfection, and PPI Assay

For PPI luminescence readout in 3D cell culture models, co-transfection was carried out in a 24-well clear flat-bottom plate with the same procedure reported for the 2D cell-based assay. At 24 h post-transfection, cells were washed with PBS 0.1 M pH 7.2, detached with Trypsin 1X, centrifugated for 5 min at 1200 rpm, and resuspended with fresh medium added with 10% (*v*/*v*) FBS charcoal stripped. Before cell seeding in 96-well microspace round-bottom cell culture plates (Elplasia^TM^ Kuraray, Tokyo, Japan), 100 µL of complete culture medium was added to each well, followed by 200 µL of cell suspension containing 3.0 × 10^4^ cells per well. Spheroid formation was obtained by incubating the plate at 37 °C and 5% CO_2_.

Brightfield images of HEK293 spheroids were used to calculate the sphericity factor (ϕ) employing ImageJ version 1.51d software to define each spheroid’s perimeter (*P*) and projected area (*A*):

(1) $$\phi \;=\;\frac{\pi x\sqrt{\left(\frac{4A}{\pi }\right)}}{P}$$

### 2.6. 2D Live Cell Imaging

HEK293 cells, grown in 2D flat-bottom clear 24-well plates for 24 h, were washed with 200 μL of PBS 0.1 M pH 7.2 and the medium was replaced with DMEM supplemented with 10% (*v*/*v*) FBS charcoal stripped, 2 mM/L-glutamine, 50 U/mL penicillin, and 50 µg/mL streptomycin. Then, cells were co-transfected with 0.25 µg pGL3-SV40-LgBiT-hAR and 0.25 µg of pGL3-SV40-hAR-SmBiT constructs using an FUGENE^®^ HD/DNA ratio of 3:1 and incubated for 48 h at 37 °C with 5% CO_2_. Before each imaging session, cell culture medium was gently removed and replaced with 200 µL PBS 0.1 M pH 7.2 and BL imaging was performed using an Olympus CL40 inverted microscope connected to an electron multiplying charge-coupled device (EMCCD) camera (ImagEM-X2, Hamamtsu). Images of the 2D cell culture were obtained by acquiring the BL signal with a 20× objective using an integration time of 5 min, at a gain set to 500, and after 30 min, adding 60 µL/well of 5× Nano-Glo^®^ Live Cell Assay System substrate signal and 40 µL of testosterone solution (100 nM final concentration) or 40 µL of ethanol solution 1% (*v*/*v*) as a control. All experiments were performed in triplicate and repeated at least three times.

## 3. Results

### 3.1. Characterization of the NanoBiT Reporter Expressed in 2D and 3D Cell Models

**Figure 2:**
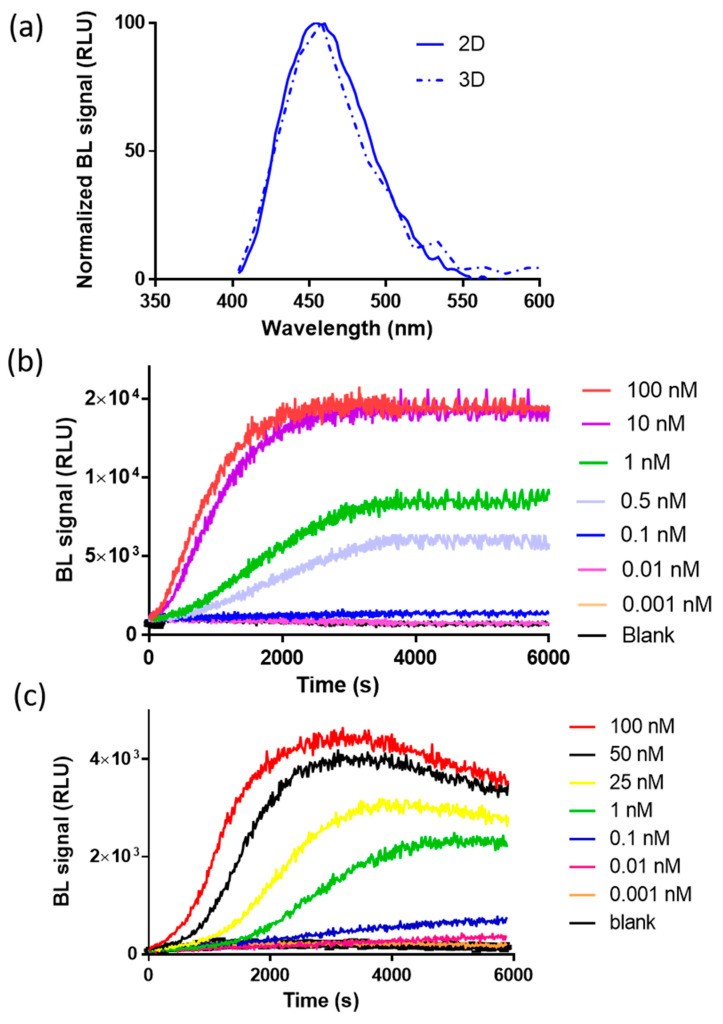
Characterization of the bioluminescence (BL) nanosensor signal in 2D and 3D cells. (**a**) Normalized emission spectra of reconstituted NanoBiT in spheroids (dotted line) and in 2D cell cultures (solid line) after the addition of 100 nM of testosterone (final concentration). Emission spectra were obtained in nonlysing conditions using the Nano-Glo^®^ Live Cell Assay System substrate; (**b**) emission kinetics of reconstituted NanoBiT in the 2D cell culture obtained in nonlysing condition after the addition of testosterone solutions (concentration range of 0.001–100 nM); (**c**) emission kinetics of reconstituted NanoBiT in HEK293 spheroids obtained in nonlysing condition after the addition of testosterone (concentration range of 0.001–100 nM).

To preliminarily investigate the suitability of complementation of split NanoLuc luciferase in 2D and 3D cell models, bioluminescence measurements were performed in nonlysing conditions using HEK293 co-transfected with pGL3-SV40-LgBiT-hAR and pGL3-SV40-hAR-SmBiT constructs and treated with testosterone at a final concentration of 100 nM. The binding of testosterone to hAR activates a series of conformational changes leading to homodimerization and complementation of the split NanoLuc luciferase. We first characterized NanoBiT re-assembly in HEK293 grown as a monolayer and in HEK293 spheroids with an average diameter of 130 ± 20 µm. This size was selected because it has been shown to be suitable for maintaining an adequate cell viability with a negligible necrotic core, of less than 2% of cells [23,24,25]. Since all BL systems require molecular oxygen, the availability of oxygen is a crucial factor in BL measurements. This is not an issue when cells are grown as a monolayer; however, it must be taken into consideration with 3D cell models. Therefore, spheroids with a maximum diameter of 150–200 µm were used. Emission spectra and kinetics were measured to highlight issues that could circumvent BL detection in 3D cultures, such as substrate and oxygen availability issues at the core of spheroids. As expected, the emission spectrum of reconstituted NanoBiT did not significantly change when compared with that obtained with HEK293 monolayer cultures (Figure 2a). The maximum emission wavelength was 460 nM in 2D cultures and 458 nM in 3D cultures, with a minor broadening effect reported in the 3D format, with a bandwidth at half-maximal intensity of 53 nM vs. 75 nM obtained in 2D cultures.

BL emission kinetics in 2D and 3D cell cultures were obtained by co-transfecting 2D and 3D HEK293 cell models with plasmids, driving the expression of LgBiT-hAR and hAR-SmBiT under the regulation of the SV40 promoter, after the addition of testosterone (concentration range from 0.001 to 100 nM), showing a glow-type emission with a maximum BL signal obtained at approximately 30 min after substrate addition in the 2D cell culture (Figure 2b); the maximum signal was obtained at different times, depending on the testosterone concentration, ranging from 50 to 65 min in 3D cell culture models (Figure 2c). We identified an optimal acquisition time window between 40 and 60 and 50 and 65 min for 2D and 3D cell models, respectively.

**Figure 3:**
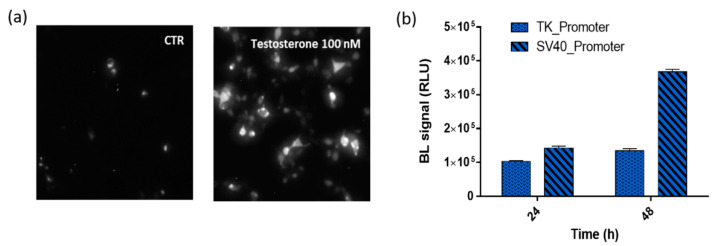
BL nanosensor expression. (**a**) BL imaging of the Human Embryonic Kidney (HEK293) monolayer culture (20× objective, 5-min acquisition) co-transfected with pGL3-SV40-LgBiT-hAR and pGL3-SV40-hAR-SmBiT and treated, 48 h post-transfection, with 100 nM testosterone solution or medium only as a control. (**b**) BL signals obtained from HEK293 co-transfected with the two chimeric proteins LgBiT-hAR and hAR-SmBiT under the regulation of thymidine kinase (TK) or simian virus 40 (SV40) promoters at 24 and 48 h post-transfection after the addition of 100 nM testosterone.

To fully confirm the reconstitution of NanoBiT after testosterone addition, BL imaging of HEK293 cell (8.0 × 10^4^ cells per well) monolayers co-transfected with plasmids driving the expression of LgBiT-hAR and hAR-SmBiT was performed (Figure 3a). Cells were grown as a monolayer and 48 h post-transfection, were imaged using a 20× objective to identify single cells and allowing, by BL imaging, the direct visualization of AR dimerization in living cells at a subcellular level. Due to the lower BL intensities and blurry signals in 3D cell models, BL imaging performed in HEK293 spheroids did not provide additional information (data not shown).

### 3.2. Comparison of Chimeric Protein Expression under TK and SV40 Promoters

Preliminary co-transfection studies were carried out to identify the optimal transfection conditions and to optimize the transfection efficiency with suitable amounts of LgBiT-hAR and hAR-SmBiT chimeric proteins. Different combinations were tested and using 50% of pGL3-SV40-LgBiT-hAR and 50% of pGL3-SV40-hAR-SmBiT provided the strongest signals and widest dynamic range. We investigated the suitability of using a strong SV40 and weak TK promoter and different post-transfection incubation times were evaluated (24 and 48 h).

The comparison of the BL emissions obtained from the two chimeric proteins LgBiT-hAR and hAR-SmBiT under the regulation of the TK and SV40 promoters is shown in Figure 3b, where, at 24 and 48 h post-transfection, the TK/SV40 ratio is 0.75 and 0.38, respectively. Moreover, by comparing the same promoter at different post-transfection incubation times, the TK and SV40 promoters show a 48/24 h ratio of 1.4 and 2.66, respectively. These results confirmed that the brightest BL signal was obtained when using plasmids in which the two chimeric proteins LgBiT-hAR and hAR-SmBiT were under the regulation of the SV40 promoter.

### 3.3. 3D Bioluminescent Assay for Androgen Receptor Activation Monitoring

**Figure 4:**
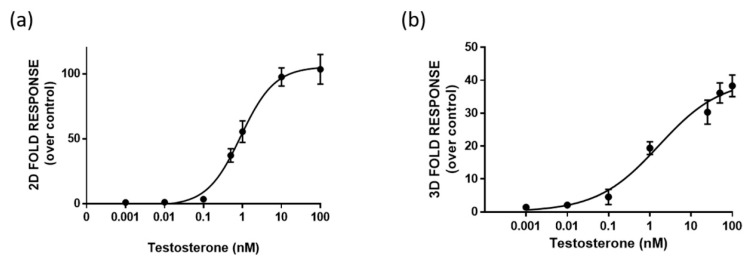
Dose-response curve for testosterone obtained in 2D cell culture (**a**) and 3D cell culture models (**b**). HEK293 cells were co-transfected with LgBiT-hAR and hAR-SmBiT under the regulation of the SV40 promoter and treated at 48 h with testosterone. BL measurements were obtained after the addition of Nano-Glo^®^ Live Cell Assay System substrate. The results are expressed as the fold response over basal control activity. Data are the mean ± s.d. of at least three independent experiments, each performed in triplicate.

One-day-old HEK293 spheroids, previously co-transfected in the 2D monolayer with plasmids driving the expression of LgBiT-hAR and hAR-SmBiT under the regulation of the SV40 promoter, were treated with different concentrations of testosterone (concentration range of 0.001–100 nM). HEK293 spheroids with a uniform size and shape exhibited BL emission that increased with the testosterone concentration in a dose-dependent matter. We chose to treat 1-day-old already-formed aggregates with a mean diameter of 130 ± 20 µM, in order to visualize the cell response after treatment, based on NanoBiT re-assembly in the presence of testosterone. Dose-response curves for testosterone and the calculated half maximal effective concentrations (EC_50_) were obtained with both 2D cell cultures and spheroids, obtaining EC_50_ values of 0.86 ± 0.02 and 1.7 ± 0.3 nM, respectively (Figure 4a,b).

A lower limit of detection (LOD) of 4 pM, calculated as the concentration that corresponds to the blank plus three times the standard deviation, was obtained in 3D spheroids in comparison to that obtained with 2D cells (40 pM).

**Figure 5:**
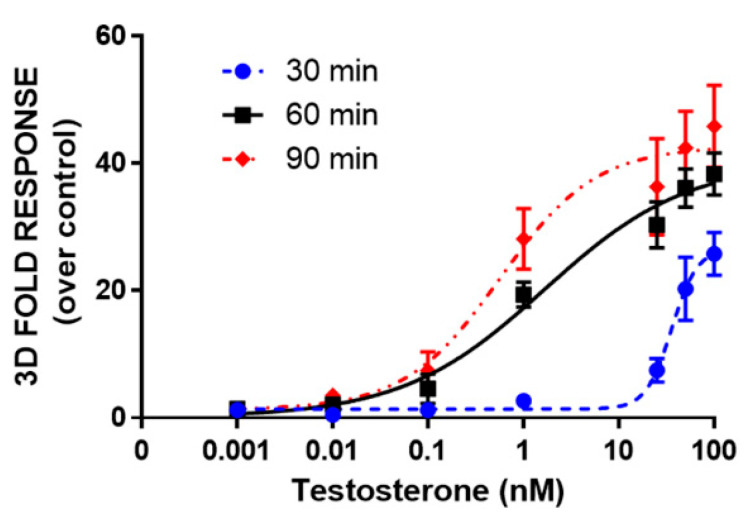
Dose-response curve for testosterone obtained in 3D cell culture models. HEK293 cells were co-transfected with LgBiT-hAR and hAR-SmBiT under the regulation of the SV40 promoter and treated at 48 h post-transfection with testosterone (concentration range of 0.001–100 nM). BL measurements were obtained after the addition of Nano-Glo^®^ Live Cell Assay System substrate and analyzed the BL signals at 30, 60, and 90 min. Data are the mean ± s.d. of at least three independent experiments, each performed in triplicate.

Moreover, dose-response curves for testosterone were obtained in HEK293 spheroids by analyzing BL signals at 30, 60, and 90 min (Figure 5). LODs of 1.2 nM, 4 pM, and 1 pM were obtained at 30, 60, and 90 min, respectively. EC_50_ values were 36 ± 2, 1.7 ± 0.3, and 0.6 ± 0.1 nM, calculated at 30, 60, and 90 min, respectively. The light signal was proportional to the concentration of testosterone at 60 and 90 min, showing a linear range from 0.007 to 100 and 0.008 to 25 nM, respectively. The response was reproducible with an intra-assay variability of 18% and an inter-assay variability of 25%. This variability could be associated with transient transfections and it could be reduced by employing stable transfection [26].

## 4. Discussion

A novel intracellular nanosensor was developed based on the complementation of split NanoLuc luciferase. Thanks to the NanoBiT high BL signal, low self-assembly affinity, and high signal to noise ratio, the expression of fusion proteins can be kept at physiological levels, thus providing an efficient tool for quantitatively investigating PPI under physiological conditions [27]. We developed an assay to monitor homodimerization of the human androgen receptor in real time and in 3D cell models using the NanoBiT split-luciferase system. The androgen receptor is an important therapeutic target that plays a pivotal role in mediating several diseases, including prostate cancer and male infertility. Therefore, the availability of methods for identifying new compounds with androgenic activity is of vital importance. Most of the assays and biosensors previously reported by us and others rely on reporter gene technology, exploiting BL and fluorescent reporters under the control of the androgen response element (ARE) in mammalian and yeast cells [28,29]. Although these assays are generally very sensitive, they do not provide an accurate picture of intracellular dynamics. Conversely, PCA enables the spatiotemporal dynamics of receptor activation to be investigated. Generally, all these assays are performed with conventional 2D cell cultures and are considered an important pillar of the drug discovery process. Sometimes, these tests provide misleading and nonpredictive data on in vivo responses.

To overcome many of the limitations of traditional 2D cell culture systems, the 3D cellular system was selected to reproduce cell–cell and cell–matrix interactions, intra- and inter-cellular signalling networks, and diffusion/transport conditions, which are important for differentiation, proliferation, and various cellular functions.

We chose the HEK293 cell line for transient co-transfection with plasmids driving the expression of similar expression levels of LgBiT-hAR and hAR-SmBiT under the regulation of the SV40 promoter, which allowed the strongest signals and widest dynamic range (Figure 3b). The SV40 promoter was selected to increase chimeric receptor expression and the ratio of recombinant constructs with respect to endogenous receptors. This reduces competition for dimerization with native androgen receptors, which are present at very low levels in HEK293 cells [30]. NanoBiT reconstitution was fully confirmed by BL imaging in the HEK293 monolayer with the direct visualization of hAR dimerization in living cells at a subcellular level (Figure 3).

To investigate the suitability of the novel intracellular nanosensor in 3D cell models, firstly, we characterized NanoBiT re-assembly in HEK293 grown as monolayer and in HEK293 spheroids with an average diameter of 130 ± 20 µM. The spheroid size selected is able to maintain an adequate cell viability with a negligible necrotic core, of less than 2% of cells [23,24,25] and to obtain a sufficient oxygen availability, which is essential for BL measurements. After the addition of testosterone, both 2D and 3D culture systems showed similar emission spectra and glow-type emission kinetics, with maximum BL signals at 30 and from 50 to 65 min, respectively (Figure 2b,c). This is partially due to the bioavailability of testosterone and the longer time required to penetrate the spheroid in depth, so the major contribution of BL emission will derive from external cell layers. Interestingly, a decline in the BL signal was observed after approximately one hour in 3D models, probably due to substrate inactivation. In addition, oxygen availability represents a critical factor of the cell microenvironment; as previously reported, firefly luciferin-luciferase systems exposed to hypoxic conditions (near 0% O_2_) resulted in a 3.4-fold reduction of total photon flux when compared to normoxia (21% O_2_). This decrease derives from the requirement of molecular oxygen for the oxidation of firefly luciferin. Moreover, in an ATP-dependent luciferase-catalyzed reaction, this decrease was also related to a reduction in the intracellular ATP content under hypoxia [31]. For this reason, ATP-independent luciferases, such as NanoLuc, could be more advantageous than firefly luciferases for applications in 3D cell models.

To evaluate the feasibility of using 3D bioluminescence spheroids of HEK293 for improving the predictivity of the method [32], we developed a 3D cell-based assay to monitor homodimerization of the human androgen receptor in real time using the NanoBiT split-luciferase system. To the best of our knowledge, no PCA assays have been reported in spheroids or 3D cell models. Therefore, such implementation is also highly valuable for other targets and sensor formats. Compared to a PCA reported by Kim et al., relying on the intramolecular complementation of split click beetle luciferase with an LOD for testosterone of‚ ~10^−5^ M [16], both the assays in 2D and 3D cells provided a higher sensitivity, with LODs of 40 and 4 pM, respectively. The lower LOD obtained with 3D cell models can be partially explained by the formation of spheroid structures in which cells grow in layers, similar to the in vivo condition, thus replicating not only the cellular topology, but also gene expression, metabolism, and signaling.

Moreover, the LOD obtained for the 3D cell culture system is one order of magnitude lower than that previously reported in yeast reporter assays for androgenic compounds [28] and two orders of magnitude lower than mammalian cell-based assays [26], which require longer incubation times (18 h). Considering its low limit of detection and rapidity, the developed assay could be considered a valuable alternative for the predictive screening of androgenic compounds.

The analytical performance of this 3D assay supports its use for clinical applications, i.e., to monitor total blood androgenic activity during testosterone replacement therapy [33,34]. Physiological total serum testosterone levels range from 280 to 1100 ng/dL (corresponding to 9.71–38.17 nM) for adult males; these concentrations are fully covered by the working range of the reported method. As previously demonstrated [35,36], bioassays relying on androgen receptor activation may be employed as sensitive tools to detect androgenic activity in clinical samples (e.g., plasma and urine). Moreover, these assays have the potential to detect the presence of unknown androgenic substances and identify samples that need to undergo confirmatory analysis. This possibility opens new applications for antidoping screenings.

## 5. Conclusions

We developed a new intracellular nanosensor that exploits intermolecular NanoLuc complementation for the fast and sensitive detection of androgenic-like compounds. Two chimeric proteins with a split bioluminescent reporter (SmBit and LgBit) fused with an hAR receptor were expressed in HEK293 cells. The feasibility of monitoring receptor dimerization in real time was also demonstrated with bioluminescence single cell imaging. To assess the suitability of this nanosensor in 3D models, HEK293 spheroids were engineered to express the two chimeric constructs and an adequate analytical performance was achieved. After further characterization, this nanosensor could find application not only in drug screening, but also for the rapid detection of androgen-like chemicals, for example, for monitoring endocrine disrupting compounds in food and environmental samples. To expand the applicability of the sensor, future work will involve an investigation of anti-androgenic molecules’ effect on the nanosensor and the analysis of complex matrices.

## Data Availability

Data sharing is not applicable to this article.

## Supplementary Materials

The following are available online at https://www.mdpi.com/1424-8220/21/3/893/s1. Figure S1: Representative plasmid maps for the vectors pGL3-Control-SV40-hAR-SmBiT and pGL3-Control-SV40-LgBiT-hAR.
