# Supplementary material for: A Genetically Encoded Bioluminescence Intracellular Nanosensor for Androgen Receptor Activation Monitoring in 3D Cell Models
Source: Sensors (Basel). 2021 Jan 29;21(3):893. doi: 10.3390/s21030893 (PMC7865915; doi:10.3390/s21030893)

Supplementary Materials

Genetically encoded bioluminescence intracellular nanosensor for androgen receptor activation monitoring in 3D cell models

Maria Maddalena Calabretta[1,2], Antonia Lopreside[1,2], Laura Montali[1,2], Luca Cevenini[1], Aldo Roda[1,3], Elisa Michelini*[1,2,3,4]

[1]Department of Chemistry "Giacomo Ciamician", University of Bologna, Via Selmi 2, 40126 Bologna, Italy

[2] Center for Applied Biomedical Research (CRBA), University of Bologna, Italy

[3]INBB, Istituto Nazionale di Biostrutture e Biosistemi, 00136 Rome, Italy

[4]Health Sciences and Technologies-Interdepartmental Center for Industrial Research (HST-ICIR), University of Bologna, via Tolara di Sopra 41/E 40064, Ozzano dell'Emilia, Bologna, Italy

Figure S1: Representative plasmid maps for the vectors pGL3-Control-SV40-hAR-SmBiT and pGL3-Control-SV40-LgBiT-hAR.

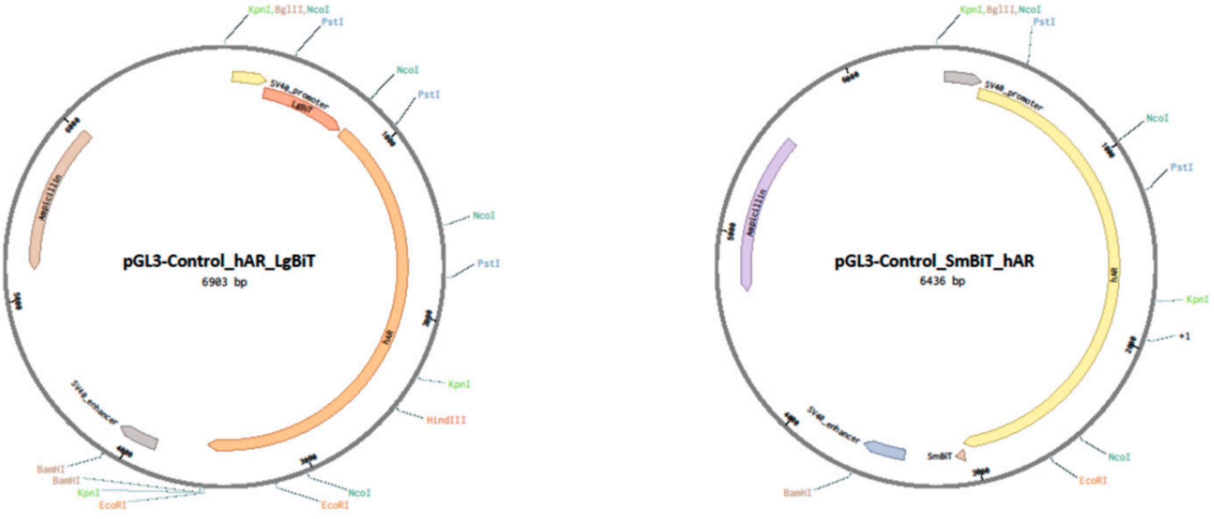

Supplement: Supplementary file 1 [file sensors-21-00893-s001.pdf]
